# Effect of sex on glomerular filtration rate in programmed rats by prenatal dexamethasone

**DOI:** 10.14814/phy2.14154

**Published:** 2019-06-26

**Authors:** Jyoti Jain, Susan K. Legan, Issa Alhamoud, Jyothsna Gattineni, Michel Baum

**Affiliations:** ^1^ Department of Pediatrics University of Texas Southwestern Medical Center at Dallas Dallas Texas; ^2^ Department of Internal Medicine University of Texas Southwestern Medical Center at Dallas Dallas Texas

**Keywords:** Glomerular filtration rate, hypertension, prenatal programming, sex

## Abstract

We have previously demonstrated that dexamethasone administered to pregnant rats during specific times during gestation results in a reduction in glomerular number and hypertension in offspring at 2 and 6 months of age. In this study, we examined the effect of prenatal dexamethasone administered daily on days 15 and 16 of gestation in male and female offspring after 1 year of age on glomerular filtration rate. The prenatal dexamethasone male group had a higher systolic blood pressure than the vehicle male group. Females had lower systolic blood pressures than the males and prenatal dexamethasone did not affect blood pressure in female offspring. Prenatal dexamethasone resulted in a reduction in glomerular filtration rate in male but not in female rats. When corrected for body weight, the control male rats had a lower glomerular filtration rate than the control female rats. Males had greater protein excretion than females and prenatal dexamethasone increased the protein excretion only in male rats. Glomerulosclerosis was also greater in male rats than females but was not affected by prenatal dexamethasone. In summary, male rats appear to have evidence of a decline in glomerular filtration rate after 1 year of age and prenatal dexamethasone programs an accelerated decline in glomerular filtration rate in male but not in female offspring.

## Introduction

There is substantive evidence that low birth weight impacts glomerular endowment. In an autopsy study of African Americans and Caucasians in the Southeast United States, the number of glomeruli ranged from about 200,000 to 1.8 million and there was a direct relationship between glomerular number and birth weight (Hughson et al. 2003). In addition, there was also an inverse relationship between glomerular volume and glomerular number consistent with a low nephron number resulting in compensatory glomerular hypertrophy (Hughson et al. 2003). Birth weight affected the number of glomeruli in both sexes but only in males was the correlation between birth weight and the number of glomeruli significant (Hughson et al. 2003). Glomerular number and volume have been estimated in human neonates born after 36 weeks gestation, a time when nephrogenesis is complete, who died from nonrenal causes before 2 weeks of age (Manalich et al., 2000). There was a direct relationship between birth weight and nephron number and an inverse relationship between nephron number and glomerular volume (Manalich et al., 2000).

A paucity of glomeruli at birth is likely a harbinger for progressive renal disease in later life. As postulated by Brenner et al., a reduced nephron number will result in a compensatory increase in glomerular capillary pressure and an increase in glomerular volume to maintain the glomerular filtration rate (Brenner and Chertow 1994). However, this increase in single‐nephron glomerular filtration rate is maladaptive in the long run leading to focal and segmental glomerulosclerosis which is likely the result of sustained glomerular capillary hypertension. The loss of glomeruli will result in a vicious and accelerating cycle of glomerular loss and a reduction in total renal glomerular filtration rate via the above mechanism. While there has been some discrepancy in studies examining the effect of birth weight on chronic kidney disease, a meta‐analysis examining 18 studies found that low birth weight resulted in an increase in albuminuria, chronic kidney disease, and end‐stage renal disease (White et al. 2009). Overall, there is a 70% increased risk of developing chronic kidney disease if you are of low birth weight compared to those with a normal birth weight (White et al. 2009).

Whether there is a sex difference in the susceptibility to develop a decline in renal function in patients who are of low birth weight is unclear. In a study examining young adults between 20 and 30 years of age who were born with low birth weight, there was a small decrease in estimated glomerular filtration rate and the effect was greater in men than women (Hallan et al. 2008). A study comparing 26 ± 1.9‐year‐old females who were appropriate for gestational age to those born before 32 weeks or born at term but having a birth weight <2600 g found that neither prematurity nor small for gestational age affected glomerular filtration rate (Kistner et al. 2000). The Helsinki Birth Cohort Study which examined adults born between 1924 and 1944, found that small for gestational age was a risk factor to develop chronic kidney disease in men but not women, while both sexes were at risk for developing chronic kidney disease if they were premature (Eriksson et al. 2018). Assessment of the Medical Birth Registry of Norway disclosed that a birth weight of less than the tenth percentile was a risk factor for developing end‐stage renal disease in both men and women, but males had a 1.4‐fold greater relative risk than females (Vikse et al. 2008). When corrected for gestational age only, men born with a birth weight less than the tenth percentile had an increased risk of end‐stage renal disease (Vikse et al. 2008). However, other studies have found that both men and women who were small for gestational age at birth are at risk for end‐stage renal disease, but the effect of low birth weight was statistically significant only for women (Lackland et al. 2000). Thus, the effect of sex on the development of chronic kidney disease in patients who were born of low birth weight in these retrospective studies is not consistent.

Rodents exposed to prenatal insults such as a maternal low‐protein diet, prenatal glucocorticoids, or uterine insufficiency have demonstrated that prenatal insults result in a reduction in glomerular number (Moritz et al. 2009; Ortiz et al. 2001, 2003; Vehaskari et al. 2001; Wlodek et al. 2007). We have previously found that in 6‐month‐old rats whose mothers were administered dexamethasone on days 15 and 16 of gestation, only the males developed hypertension and there was no decrease in glomerular filtration rate at 6–9 months of age (Ortiz et al. 2003). We recently found that male rats whose mothers were fed a 6% protein diet during the last half of gestation had a reduction in glomerular filtration rate when measured at 1.5 years of age (Lozano et al. 2015; Mansuri et al. 2017). We hypothesized that prenatal dexamethasone had a progressive effect on glomerular filtration rate that would be apparent in older rats. The purpose of this study was to examine if prenatal dexamethasone results in a decline in glomerular filtration rate after 1 year of age and to determine if females were protected from a reduction in glomerular filtration rate by prenatal programming.

## Methods

### Animals

Pregnant Sprague–Dawley rats were injected with 0.2 mg/kg body weight dexamethasone or vehicle on days 15 and 16 of gestation. We have previously shown that administration of dexamethasone at this time of gestation results in hypertension (Ortiz et al. 2003). Rats were provided free access to food and water. There were no more than three rats of each sex used in these studies from any litter. There were a total of 6 litters in the vehicle‐treated and 6 litters from the dexamethasone‐treated rats in these studies. From some litters, only one or two rats of a given sex were used. All the rats were euthanized after the measure of GFR or by 15 months of age. The IACUC of the University of Texas Southwestern Medical Center approved of these studies.

### Glomerular filtration rate

Glomerular filtration rate was measured in male and female rats in the vehicle and dexamethasone groups at 15 months of age. We used the same methodology as we have described previously (Lozano et al. 2015; Mansuri et al. 2017; Ortiz et al. 2003). Briefly, rats were anesthetized with an intraperitoneal injection of Inactin (10 mg/100 g body weight). The rats were then placed on a heated table to maintain their body temperature at 37°C. A PE10 intravenous line was placed in the femoral vein to administer ^3^H‐methoxy inulin. A tapered PE20 catheter was placed in the carotid artery for blood sampling. A tracheostomy was performed and PE240 catheter was placed in the trachea. Finally, a PE20 catheter was inserted in the bladder and 6‐O silk was used to secure the bladder catheter and prevent leakage of urine.

^3^H‐methoxy inulin was dissolved in normal saline at approximately 40 *μ*Ci/mL. A bolus of 0.6 mL/100 g body weight was administered over the first hour followed by a constant infusion of 0.36 mL/100 g body weight. After at least 1 h of incubation at the constant infusion rate, the clearance experiment was started. There were five 30‐min collections of urine with mid‐point collections of blood. Fifty microliter samples of serum and urine were used to assess ^3^H‐methoxy inulin counts using a Tri‐Carb 2100 TR liquid scintillation analyzer, PerkinElmer (Shelton, CT).

### Blood pressure

Systolic blood pressure was measured when the rats were 13 months of age. The investigator measuring the blood pressure (J.J.) was blinded as to whether the rat was from the vehicle or the dexamethasone group. Rats were trained for four consecutive days by placing them in a large Lucite container and inflating a cuff around its tail several times (Mansuri et al. 2015; Mizuno et al. 2014, 2013). We then measured the blood pressure on the fifth day using a CODA Blood Pressure Non‐Invasive Pressure Analyzer (Kent Scientific Corporation, Torrington, CT). The CODA Blood Pressure System uses volume pressure recording which has been shown to correlate with measurements made using telemetry (Feng et al. 2008). The mean of at least five successful readings was used as the blood pressure for the rat.

### Serum creatinine and urine collection

Rats were placed in metabolic cages at 14 months of age. They were given free access to food and water. After 24 h of acclimation, a 24‐h urine collection was started. The Bradford assay was used to measure urine protein (Bio‐Rad Laboratories; Hercules, CA). Urine albumin was measured with a rat urinary albumin enzyme immunoassay kit (Nephrat; Exocell; Philadelphia, PA). Serum and urine creatinine were assayed using capillary electrophoresis.

### Measurement of interstitial fibrosis and glomerular injury

After measurement of glomerular filtration rate, the kidneys were assessed for interstitial fibrosis and glomerular injury by a blinded investigator (IA). To assess fibrosis, kidney slices were stained with picrosirius red. Slides were photographed using an Axioplan‐2 Zeiss microscope with a Zeiss Axiocam MRC3 camera (Carl Zeiss Thornwood, NY). At least 10 images of cortex and 10 images of outer medulla were photographed and the images were analyzed using NIS‐Elements BR 3.2 software to quantify fibrosis (Chen et al. 2011). The total field (minus glomeruli and large blood vessels) was examined at 300‐fold magnification (Oda et al. 2001). The percent of the image analyzed that was stained with picrosirius red was compared between the four groups.

Glomerular injury was assessed for the degree of mesangial matrix expansion and glomerulosclerosis using the same definition and scale described by Raij et al. at 300‐fold magnification (Raij et al. 1984). For these studies, the slides were stained with periodic acid‐Schiff. Glomerular mesangial matrix expansion was assessed as the amount of periodic acid‐Schiff staining (graded 0–4). The percentage of the glomerulus sclerosed was scored 0–4 based on the fraction of glomerular involvement. Twenty glomeruli were assessed on each slide and the average score for the slide was used as the score for that slide. The average score for each slide was multiplied by 100 to give a score of 0 to 400 for matrix expansion and glomerulosclerosis.

The amount of collagen was also determined in the kidneys from rats after measurement of glomerular filtration rate. The collagen content was determined by measuring hydroxyproline. Approximately, 10 mg of kidney cortex was weighed and assayed for hydroxyproline using a Hydroxyproline Colorimetric Assay Kit (BioVision Incorporated, Milpitas, CA) per manufacturer's instructions. Homogenized tissue was hydrolyzed with 6N hydrochloric acid at 120°C for 3 h. Samples were evaporated to dryness and collagen abundance was extrapolated from the hydroxyproline content assuming that collagen contains 12.7% hydroxyproline by weight. Results were expressed as *μ*g collagen/mg kidney.

### Chemicals

Chemicals were purchased from Sigma Chemical Company unless otherwise specified (St. Louis, MO).

### Statistics

All data are expressed as the mean ± the standard error of the mean. We compared the four groups using two‐way analysis of variance with a Student‐Newman‐Keuls post hoc analysis.

## Results

**Figure 1:**
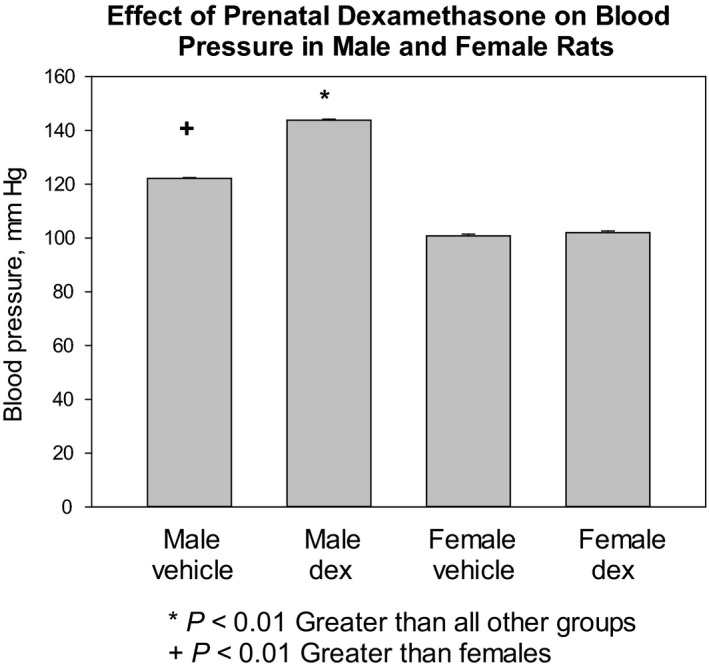
Effect of prenatal dexamethasone on systolic blood pressure in male and female rats. Blood pressure was higher in male rats than female rats and prenatal dexamethasone‐treated male rats had higher blood pressure than all other groups. There were at least 14 rats in each group.

In the first series of experiments, we measured systolic blood pressure by tail cuff in trained rats at 13 months of age. The results are shown in Figure 1. The female vehicle and dexamethasone groups had comparable blood pressures which were significantly lower than the male groups. Prenatal dexamethasone males had higher blood pressures than the vehicle males and any other group. Thus, prenatal dexamethasone causes elevated blood pressure only in male rats.

**Figure 2:**
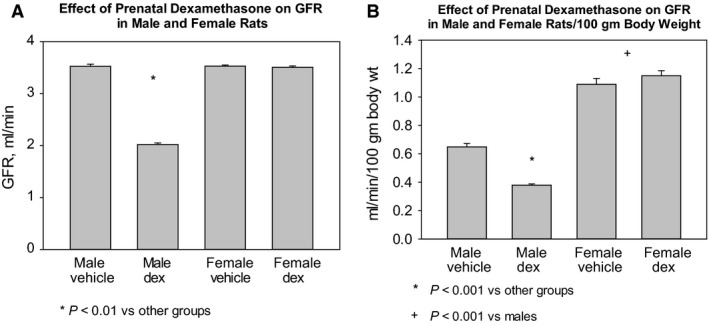
Effect of prenatal dexamethasone on glomerular filtration rate in male and female rats. Prenatal dexamethasone‐treated male rats had a significantly lower glomerular filtration rate than all other rats (A). When corrected for body weight (B), the males had a lower glomerular filtration rate than the females. The female prenatal dexamethasone and vehicle group had comparable glomerular filtration rates. The male prenatal dexamethasone group had a lower glomerular filtration rate than the male vehicle group. There were 12 rats in each group.

The effect of prenatal dexamethasone on glomerular filtration rate in male and female rats at 15 months of age are shown in Figure 2. Uncorrected glomerular filtration rates were comparable in male vehicle and female vehicle rats (Fig. 2A). There was no effect of prenatal dexamethasone on glomerular filtration rate in female rats; however, male rats whose mothers were administered dexamethasone had a lower glomerular filtration rate than the vehicle‐treated male rats. When corrected for body weight, male vehicle rats had a lower glomerular filtration rate than female vehicle rats (Fig. 2B). Prenatal dexamethasone had no effect on the glomerular filtration rate in females compared to vehicle‐treated offspring. Offspring of dexamethasone‐treated male rats had a lower glomerular filtration rate than all other groups. Thus, prenatal dexamethasone programmed a reduction in glomerular filtration rate only in male rats. When corrected for body weight, the male vehicle group had a lower glomerular filtration rate than the female vehicle group. The male dexamethasone rats also had a higher serum creatinine than the male vehicle and female dexamethasone rats (Table 1).

**Table 1:** Effect of prenatal dexamethasone on serum creatinine and urine protein and albumin excretion in 14‐month‐old‐male and female rats

	Serum Cr	Urine protein/Cr	24‐h urine protein (mg)	Urine albumin/Cr	24‐h urine albumin (mg)
Male Vehicle	0.35 ± 0.02	9.1 ± 2.2^a^	151 ± 30^a^	2.3 ± 0.5	38 ± 5^a^
Male Dex	0.51 ± 0.05^b^	18.0 ± 2.0^c^	255 ± 27^c^	4.0 ± 0.5^c^	55 ± 6^d^
Female Vehicle	0.43 ± 0.03	4.2 ± 2.1	34 ± 29	2.2 ± 0.5	17 ± 6
Female Dex	0.40 ± 0.03	4.9 ± 2.0	37 ± 28	2.8 ± 0.5	22 ± 3^d^

*N* ≥ 13 in each group.

a *P* < 0.05 males greater than females.

b *P* < 0.05 versus Male Vehicle and Dex Female.

c *P* < 0.05 versus all groups.

d *P* < 0.05 Dex greater than vehicle.

We next examined if prenatal dexamethasone caused an increase in urinary protein excretion compared to vehicle‐treated rats (Table 1). We examined protein excretion normalized per creatinine excretion as well as a 24‐h protein excretion. The results were the same. There was no difference in urinary protein excretion comparing the female prenatal vehicle and the female prenatal dexamethasone groups. The male vehicle group had a significantly higher rate of protein excretion than either of the female groups. The male dexamethasone group had a higher protein creatinine ratio and 24‐h protein excretion than the male vehicle group and the female groups. Urinary albumin/creatinine was higher in the dexamethasone‐treated male group than in the male vehicle group. Comparing 24‐h urinary albumin excretion, dexamethasone increased the albumin excretion and males had a greater albumin excretion than females.

**Figure 3:**
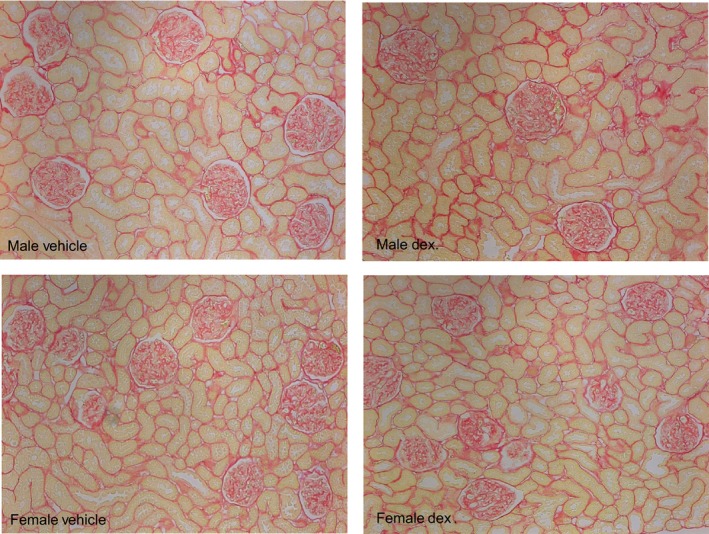
Effect of prenatal dexamethasone on renal histology.Shown are male (top) and female (bottom) renal slides stained for picosirus red at 100× magnification. Quantitation of fibrosis is shown in Table 2. As can be seen, there is little fibrosis or glomerulosclerosis in the male and female dexamethasone group (right) compared to the vehicle group (left).

Finally, we examine the histology of the kidneys looking at interstitial fibrosis and mesangial matrix expansion as well as glomerulosclerosis (Table 2 and Fig. 3). There was little interstitial fibrosis in the cortex and medulla which we have found previously in studies examining the effect of prenatal low‐protein diet on interstitial fibrosis in male rats (Lozano et al. 2015; Mansuri et al. 2017). In addition, there was no difference between the groups. Programed male rats had more renal cortical collagen content than females. Glomerulosclerosis was greater in the males than the female rats but prenatal dexamethasone did not cause a significant increase in glomerulosclerosis compared to the vehicle control. Prenatal dexamethasone females had less collagen than vehicle female offspring at 15 months of age.

**Table 2:** Effect of prenatal dexamethasone on renal collagen content, interstitial fibrosis, mesangial matrix expansion, and glomerulosclerosis in male and female rats

	*μ*g Collagen/mg tissue	Interstitial PS red (cortex)	Interstitial PS red (outer medulla)	Glomerular mesangial matrix expansion	Glomerulo‐sclerosis
Male Vehicle	7.2 ± 0.4^a^	7.0 ± 0.8%	3.8 ± 0.6%	216.6 ± 15.7	57.0 ± 11.2^a^
Male Dex	7.5 ± 0.9^a^	9.0 ± 0.7%	4.4 ± 0.5%	233.7 ± 15.5	67.0 ± 15.5^a^
Female Vehicle	6.5 ± 0.4	6.6 ± 0.8%	3.3 ± 0.5%	201.2 ± 16.1	17.0 ± 2.7
Female Dex	4.1 ± 0.3^b^	8.6 ± 0.5%	4.6 ± 0.3%	254.1 ± 21.0	31.2 ± 6.5

*N* = 12 in each group.

a *P* < 0.05 Male versus Female rats.

b *P* < 0.001 Female Dex versus Vehicle rats.

## Discussion

This study examined the effect of prenatal dexamethasone on blood pressure and glomerular filtration rate in male and female rats after 1 year of age. We demonstrate that male but not female rats whose mothers received prenatal dexamethasone on days 15 and 16 of gestation had an elevation in blood pressure, proteinuria, and a reduction in glomerular filtration rate compared to control rats. This study shows that there is a decline in glomerular filtration rate in males but not in females with prenatal programming by dexamethasone.

The effect of prenatal programming on the number of glomeruli has been studied previously but most studies have employed male rats where a reduction in glomerular number is almost universally demonstrated. The effect of sex on the number of glomeruli after a prenatal insult has yielded variable results in studies looking at rats. Woods et al. found that adult male rats whose mothers were fed an 8.5% protein diet compared to 19% in controls had a 25% reduction in glomeruli (Woods et al. 2001), while female offspring had a comparable number of glomeruli when mothers were fed either diet (Woods et al. 2005). A similar result showing a reduction in glomerular number only in male offspring was found in rats whose mothers were fed a 9% protein diet (Boubred et al. 2016). However, a more significant maternal protein restriction of 6% compared to a 20% control group resulted in a comparable reduction in glomerular endowment in both male and female offspring (Vehaskari et al. 2001). In a study where 0.25 mg/kg betamethasone was administered to pregnant rats on days 17–19 of gestation, both male and female offspring had a reduction in glomerular number comparable to the results of this study (Boubred et al. 2016). However, a lower dose of dexamethasone (0.1 mg/kg) administered daily throughout gestation resulted in a reduction in glomerular number in males but not female offspring (Martins et al. 2003). In our previous studies, we found that dexamethasone (0.2 mg/kg) on days 15 and 16 gestation induced a reduction in glomeruli but we did not analyze males from females independently (Ortiz et al. 2001, 2003). In a post hoc analysis, we reanalyzed the data and found that prenatal dexamethasone caused a 20% reduction in glomerular number in males compared to the vehicle male group (*P* < 0.001), and prenatal dexamethasone caused a 25% reduction in the females compared to vehicle (*P* < 0.001). Thus, it may be that a mild prenatal low‐protein diet or low‐dose dexamethasone may have an effect on glomerular number only in male offspring, whereas a more severe decrease in maternal protein intake or high‐dose prenatal glucocorticoids during a critical period of gestation affects glomerular endowment in both sexes.

Previous studies have examined if there was any effect of sex of the offspring on blood pressure after a maternal insult causing small for gestational age. Male and female rats whose mothers were fed a 6% protein diet during the last half of pregnancy had elevated blood pressures compared to control rats whose mothers were fed a 20% protein diet from 2 months until last measured at 10 months of age (Vehaskari et al. 2001). A less severe maternal protein diet of 8.5% during pregnancy resulted in elevated blood pressure in male but not female adult offspring (Woods et al. 2001, 2005). Woods et al. found that a more severe maternal protein restriction to 5% resulted in hypertension in both male and female offspring (Woods et al. 2004). Others have examined if there was a sex difference in blood pressure utilizing the uteroplacental insufficiency model of prenatal programming. Blood pressure was measured in male and female offspring at various times during postnatal maturation. Male offspring were hypertensive at 1, 2, 3, 6, and 12 months of age, while females were hypertensive at 1, 2, and 12 months of age and had comparable blood pressures as controls at 3 and 6 months of age (Alexander 2003; Intapad et al. 2013). Thus, postnatal age may also be a factor determining whether sex has an effect on blood pressure in programed rats. We have previously examined the effect of sex on blood pressure using this same model of prenatal programming as in this study. Male and female offspring of rats whose mother was injected with dexamethasone on days 15 and 16 of gestation had an elevated blood pressure at 2 months of age but only males were hypertensive at 6 months compared to the offspring of vehicle‐injected controls (Ortiz et al. 2001, 2003). In the current study, programed males were hypertensive at 13 months of age while females remained normotensive.

We had previously found that prenatal programming with a maternal low‐protein diet produced little evidence of interstitial fibrosis and minimal evidence of glomerular injury in male offspring over 1 year of age (Lozano et al. 2015; Mansuri et al. 2017). Similar findings were found in male and female offspring of rats whose mothers received prenatal dexamethasone in this study. Interestingly, while prenatal dexamethasone did not increase glomerulosclerosis in this study, males demonstrated a significantly higher glomerulosclerosis score than females. We had previously found that prenatal programming with a maternal low‐protein diet had no effect on protein or albumin excretion in male rats at 1 year of age. In this study, prenatal programming with dexamethasone produced different results. Compared with vehicle‐treated offspring, prenatal dexamethasone programed an increase in both urine protein and albumin creatinine ratio in male rats. The increase in 24‐h urine protein was also greater in males whose mothers were treated with dexamethasone, but the increase in 24‐h albumin excretion in dexamethasone‐treated males did not reach statistical significance. The data are consistent with an increase in proteinuria and albuminuria in males compared to females and with prenatal dexamethasone increasing protein and albumin excretion selectively in males.

Most previous studies have found no effect of prenatal programming on glomerular filtration rate (Martins et al. 2003; Ortiz et al. 2001, 2003; Woods et al. 2001). This is likely due to the fact that the reduction in glomerular filtration rate occurs in later life than studied previously. We have shown that male rats whose mothers were fed a 6% protein diet had a comparable glomerular filtration rate as those whose mothers were fed a 20% protein diet at 3 months of age (Luzardo et al. 2011), but a significant reduction when measured at ~1.5 years of age (Luzardo et al. 2011; Mansuri et al. 2017). We have previously found that prenatal dexamethasone had no effect of glomerular filtration rate when measured at 6–9 months of age (Ortiz et al. 2003). This study combined males and females. In a post hoc analysis of these previously published data, we separated males and females and found that the glomerular filtration rate was 2.73 ± 0.17 versus 3.24 ± 0.27 mL/min control and dexamethasone male rats, respectively (Ortiz et al. 2003). There was no difference in the control versus the dexamethasone female rats (2.48 ± 0.24 versus 2.34 ± 0.19 mL/min) at this age. In the present study, we also found that the glomerular filtration rate corrected for body weight was less in male controls than female controls at 15 months of age consistent with an age‐dependent decline in male rats but not female rats. This was supported by an increase in glomerulosclerosis in male rats compared to female rats as well as the higher cortical collagen content in males compared to females. This supports findings of protection from an age‐dependent decline in renal function in females as found in previous studies (Baylis 1994; Black et al. 2015). The cause for the decrease in glomerular filtration rate in males compared to females and the lower glomerular filtration rate in male dexamethasone‐treated rats is unclear. Of note, the decline in glomerular filtration rate paralleled the higher blood pressure in males versus females and the higher blood pressure in male dexamethasone‐treated rats. Thus, hypertension could be a significant contributing factor for the decline in glomerular filtration rate. The reduction of glomeruli is also a likely factor resulting in the reduction in glomerular filtration rate in the male dexamethasone group.

In conclusion, there is an increase in blood pressure and a decline in glomerular filtration rate in male rats but not in female rats of offspring that received prenatal dexamethasone. Male rats have greater glomerulosclerosis, proteinuria and when corrected for body weight, a lower glomerular filtration rate than females. In addition, the data are consistent with an age‐dependent reduction in glomerular filtration rate in males but not female rats.

## Conflict of Interest

There is no conflict of interest by any of the authors.
